# Insights from Surgically treated Post Covid Acute Invasive Fungal Rhino-Orbital sinusitis in Chandrapur Study (SPAROS): A Population Based study of Coronavirus Associated Mucormycosis (CAM) characteristics in India

**DOI:** 10.1016/j.ijregi.2022.08.005

**Published:** 2022-08-24

**Authors:** Aakash Kasatwar, Ravindra Shukla, Nivrutti Rathod, Jayshri Nandanwar, Divyangi Mishra, Akshay Dhobley

**Affiliations:** a Department of Public Health, General Hospital Chandrapur, Maharashtra, India; b Department of Endocrinology and Metabolism, All India Institute of Medical Sciences Jodhpur, Jodhpur, Rajasthan, India; c Kasatwars Dental Hospital and Implant Centre, Chandrapur, Maharashtra, India; d Department of Radiodiagnosis, SN Medical College, Jodhpur, Rajasthan, India; e Department of Oral Pathology, Maitri Dental College, Durg, Chattisgarh, India

**Keywords:** Coronavirus-associated mucormycosis (CAM), COVID-19-associated diabetes and mucormycosis (CADM), COVID-19-associated classical mucormycosis (CACM), Coronavirus-induced mucormycosis (CIM), Methylprednisolone, Epidemiology of mucormycosis, GRP78, Diabetes mellitus

## Abstract

**Introduction:**

Coronavirus disease 2019 (COVID-19)-associated mucormycosis (CAM) became a public health problem in India in 2021. However, information about the incidence, presentation and prognosis of CAM remains sparse.

**Methods:**

This study describes 100 cases from the Surgically treated Post COVID Acute invasive fungal Rhino-Orbital Sinusitis in Chandrapur (SPAROS) study, a prospective observational follow-up study of patients with CAM diagnosed in Chandrapur district, India. Two-step cluster analysis using four input variables – blood glucose on admission, diabetes status, glucocorticoid exposure and severity of COVID-19 – was used to define three distinct CAM clusters.

**Results:**

The incidence of CAM in the general population was 7.1 cases/1000 patients hospitalized with COVID-19. Steroid exposure and pre-existing diabetes were present in 76% and 55% of cases, respectively. At median follow-up of 18 days, only two deaths had been recorded, while 93 cases were stable. Glucocorticoids, particularly methylprednisolone, seemed to precipitate CAM. Admission to the intensive care unit appeared to be predictive of less extensive surgery.

**Discussion:**

Three subtypes of CAM were identified: COVID-19-associated diabetes and mucormycosis, COVID-19-associated classical mucormycosis, and COVID-19-induced mucormycosis. A CAM hypothesis was proposed based on the dynamics of severe acute respiratory syndrome coronavirus-2 and glucose regulated protein.

**Conclusion:**

The clinical characteristics, natural course and pathogenesis of CAM differ from mucormycosis in the pre-COVID era. It is hoped that this classification will be useful in CAM management.

## Highlights

• 0.08% of patients with coronavirus disease 2019 (COVID-19), mainly males, develop mucormycosis.
• Methylprednisolone, a steroid, likely precipitates COVID-19-associated mucormycosis (CAM).
• CAM is a heterogenous disease.
• There are three subtypes of CAM: COVID-19-associated diabetes and mucormycosis, COVID-19-associated classical mucormycosis, and COVID-19-induced mucormycosis (CIM).
• Treatment of each type is different.

## Introduction

Over the last 2 years, the coronavirus disease 2019 (COVID-19) pandemic has shown two distinct epidemiological waves in India (Jain et al., 2021). The first wave was followed by increased incidence of mucormycosis from October 2020 onwards. During the second wave, mucormycosis was made a notifiable disease by law, and 45,000 cases were reported from 780 districts in India (https://timesofindia.indiatimes.com/india/over-45000-cases-of-mucormycosis-reported-in-india-health-ministry-tells-rajya-sabha/articleshow/84585912.cms). However, literature on the incidence, presentation and prognosis of COVID-19-associated mucormycosis (CAM), and how it differs from non-COVID mucormycosis is sparse. This study reports CAM data from a district registry in Chandrapur, Maharashtra, which is incidentally the region where the Delta variant of severe acute respiratory syndrome coronavirus-2 (SARS-CoV-2) was first reported (Jain et al., 2021). The present study involved prospective follow-up of cases from the mucormycosis registry of Chandrapur District between 11 and 24 May 2021.

## Materials and methods

### SPAROS study

All cases of mucormycosis were reported in the district registry during the notification period. The Surgically treated Post COVID Acute invasive fungal Rhino-Orbital Sinusitis in Chandrapur (SPAROS) study was a prospective observational study of all those who developed mucormycosis and had a positive SARS-CoV-2 reverse transcription polymerase chain reaction (RT-PCR) result after 1 March 2021. A case of mucormycosis was defined as a patient with both clinical and radiological evidence of mucormycosis, along with fungal hyphae in debrided tissue on KOH mount and confirmed on biopsy. Fungal angioinvasion was assessed on histopathology slides, and confirmed by an oral pathologist. Follow-up continued until the resolution of mucormycosis or death. The SPAROS study planned to enrol patients until 31 December 2021 or discontinuation of mandatory reporting, whichever was earlier, and included a network of primary and secondary hospitals (see Supplementary Material 1) across Chandrapur district. Exclusion criteria were: organ transplantation, active malignancy, cases lost to follow-up for >1 month, and cases who did not give consent. The objective was to describe acute invasive rhino-orbital fungal infection, and factors determining morbidity and mortality. Information was collected based on standard case record forms (see Supplementary Material 2). Follow-up information for the cases was collected on 3 June 2021. Mortality/unstable/stable condition was ascertained on follow-up and recovery status was categorized as stable or unstable/dead.

### Severity of COVID-19

The guidelines of the Indian Council for Medical Research were used to clinically classify cases of COVID-19 and decide the course of management. Cases were categorized as mild, moderate or severe. The guidelines recommend home isolation for mild cases and hospitalization for moderate/severe cases (https://www.icmr.gov.in/pdf/covid/techdoc/COVID_Management_Algorithm_17052021.pdf). Cases admitted to hospital after a positive RT-PCR result (moderate/severe cases) were categorized as Group B, and cases that were not admitted to hospital after a positive RT-PCR result (mild cases) were categorized as Group A. In addition, patients requiring oxygen in home isolation, deemed hypoxaemic, were included in Group B, as a number of moderate/severe cases of COVID-19 had to have home care due to the lack of hospital beds. This binary classification effectively separated the subjects into those with hypoxaemia and those without hypoxaemia. The computed tomography (CT) severity score, calculated from chest CT during COVID-19, is a widely used indicator of the severity of COVID-19 (Saeed et al., 2021). The CT severity scores of cases in this study were obtained from records (see Supplementary Material 2).

### Postoperative morbidity

The length of hospitalization is commonly used as an indicator of morbidity (Makino et al., 2018). However, in the prevailing circumstances, the length of hospitalization was more likely to be influenced by the use/availability of intravenous amphotericin B infusion or oral posaconazole rather than general condition. In contrast, a surgeon is likely to perform surgery depending on spread and disease burden. Also, the extent of surgery, as well as the requirement for repeated surgeries, is a strong predictor of morbidity in the head/neck/face region (Wu et al., 2018). As such, extent of surgery was used as a surrogate for mucormycosis morbidity in the present study. A procedure was categorized as ‘extended’ if partial or total maxillectomy, ethmoidectomy, mandibular surgery, tooth extraction, repeated surgery or orbital surgery was undertaken. All other types of surgery were categorized as ‘simple’.

### Mucormycosis incubation period

The date of the first SARS-CoV-2 RT-PCR-positive result and the date of admission for mucormycosis were used to calculate the CAM ‘incubation period’. If this period was <10 days, the case was classified as active COVID-19.

### Hyperglycaemic categorization

Diabetes was defined as: pre-existing, if the patient had a history of hyperglycaemia or was receiving antidiabetic medication; COVID-19-related, if hyperglycaemia manifested during or after onset of COVID-19 symptoms; and no diabetes, if the patient did not have any history of hyperglycaemia and was not receiving antidiabetic medication.

### Baseline COVID-19 epidemiological data

The number of RT-PCR-confirmed cases of COVID-19 from 1 March to 31 May 2021 was determined from the district's official bulletin. In addition, the total number of hospitalizations due to COVID-19 during the period was determined (https://covid19.nhp.gov.in). Due to partial/total lockdown during the period, there was less movement of people out of Chandrapur district. As such, this number was taken as the denominator to calculate the incidence of mucormycosis per 1000 patients hospitalized with COVID-19. In addition, blood glucose levels at admission for all cases of COVID-19 were obtained from the COVID Care Centre Chandrapur from 11 to 31 May 2021. These data were used to compare blood glucose levels at admission with those of patients with CAM. In total, the blood glucose levels at admission of 884 patients were de-identified. This study was approved by IEC vide GMCC/PSM/341/2021.

### Statistical analysis

Numerical variables are expressed as mean +/- 2 standard deviations (SD). Pearson's correlation and Spearman's correlation were used for parametric and non-parametric data, respectively. One-way analysis of variance was used to determine whether steroid exposure had any effect on incubation period. Tukey's honest significant difference (HSD) was used post-hoc to find differences between the groups. Binary logistic regression was used to identify factors predicting simple or extended surgery. The factors analysed were: blood glucose at presentation, intensive care unit (ICU) admission during COVID-19, presence of diabetes, presence of hypertension/CAD, history of glucocorticoid exposure, type of steroid exposure, and incubation period. As most variables were nominal, two-step cluster analysis was used to determine CAM subtypes, predefining cluster quality ≥0.5 (corresponding to ‘good fit’), importance of each input factor ≥0.4, and a maximum of four clusters. The following inputs were selected based on clinical relevance: blood glucose at admission, diabetes history (previous, diagnosed during COVID-19, or no diabetes), steroid exposure, and severity of COVID-19. One-way analysis of variance was used to compare mean blood glucose level at admission for each of the clusters with non-CAM subjects, and Tukey HSD was used for post-hoc analysis. SPSS Version 21 (IBM Corp., Armonk, NY, USA) was used for statistical analysis.

## Results

**Figure 1:**
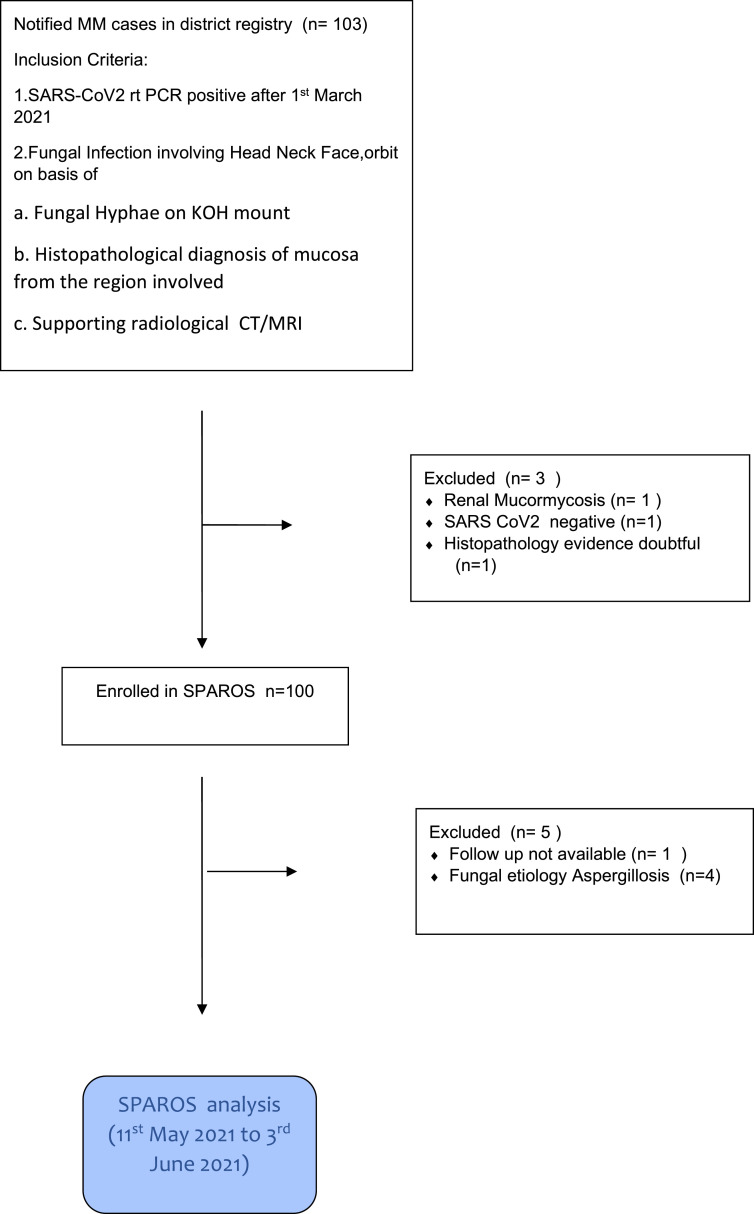
Study flowchart. MM, mucormycosis; SARS-CoV-2, severe acute respiratory syndrome coronavirus-2; RT-PCR, reverse transcription polymerase chain reaction; CT, computed tomography; MRI, magnetic resonance imaging.

In total, 100 cases were enrolled in this study between 11 and 24 May 2021 (Figure 1). The majority of cases (*n*=68) were contributed by the only tertiary hospital in the district (Government Medical College and Civil Hospital Chandrapur). The remaining 32 cases were contributed by 12 hospitals from across the district (see Supplementary Material 1). During this period, 52,648 RT-PCR-confirmed cases of COVID-19 were recorded, and the total number of patients hospitalized with COVID-19 was 13,360. The incidence of CAM, thus calculated, was 23.37/10,000 RT-PCR positive cases. The incidence of CAM was 7.1/1000 patients hospitalized with COVID-19.

The mean age of patients with CAM was 49.3 (SD 11.1) years, and the majority were male (68/96; female 28/96). Nineteen subjects had active COVID-19. All cases had maxillary sinus involvement, 68 had involvement of the ethmoid and sphenoid sinuses, and 24 had jaw and mandibular involvement. Only four cases had orbital involvement, and two of these required exenteration. There were no cases of rhino-orbito-cerebral mucormycosis or pulmonary mucormycosis.

**Figure 2:**
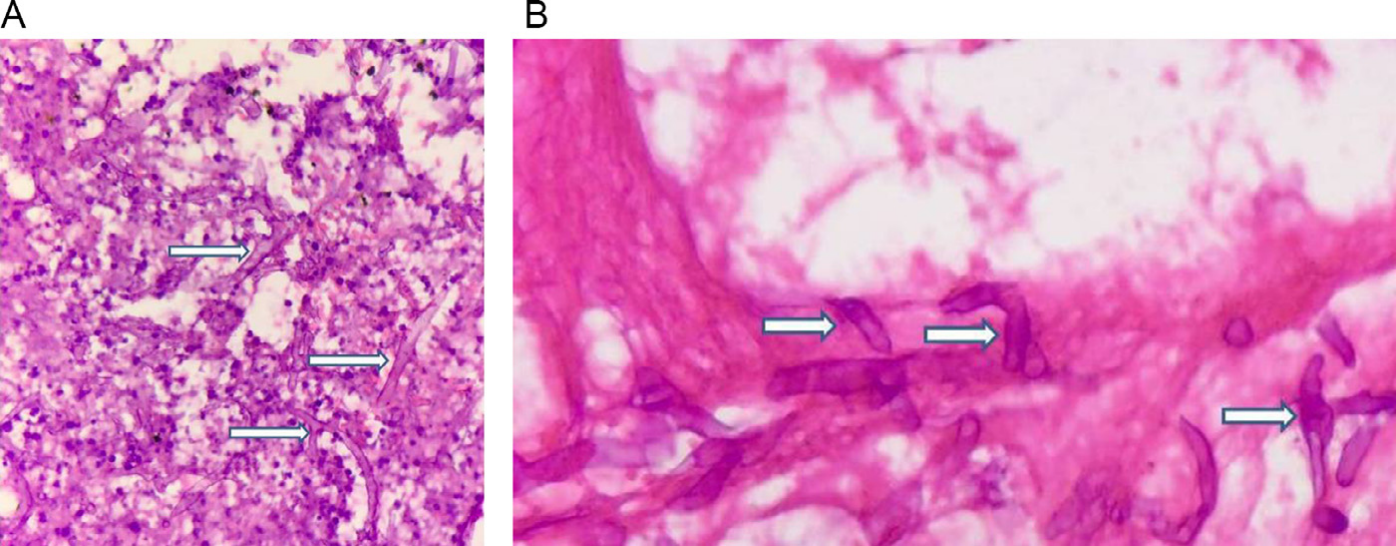
Biopsy slide of *Mucorales spp.* (A) H&E-stained section of sinus mucosa (original magnification x40) showing thick long filamentous aseptate hyphae scattered in the connective stroma. These hyphae are 12–14 microns thick and branch in obtuse angles. The stroma also have necrotic areas, chronic inflammatory cell infiltrate and dilated capillaries. (B) PAS-positive sinus mucosa (original magnification x40) showing *Mucor* spp. fungal hyphae in connective tissue stroma. Hyphae are thick aseptate, and branch at right angles. The surrounding stroma is fibrocellular.

**Figure 3:**
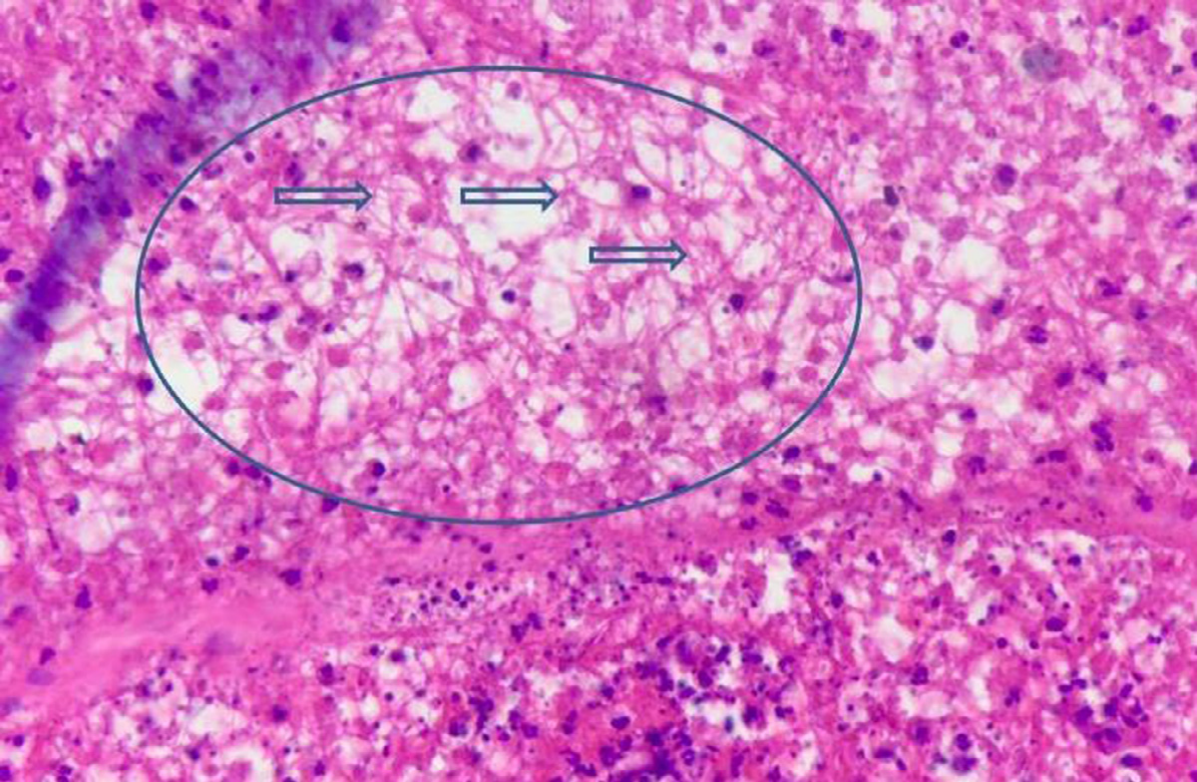
Biopsy slide of *Aspergillus* spp. H&E-stained section of sinus mucosa (original magnification x40) showing thin filamentous aseptate hyphae, 3–5 microns in diameter, which branch with acute angles, scattered in the stroma, along with lymphocytes, plasma cells, collagen fibres and fibroblasts.

Headache was the most common presenting complaint, affecting 58% of patients, followed by oral complaints such as toothache and gum swelling. Fever was conspicuous by its absence in all but four subjects (Table 1). Post-surgical biopsy revealed *Mucorales* spp. to be causative in 96 subjects and *Aspergillus* spp. to be causative in four subjects (Figure 2, Figure 3). None of the subjects had a clinical diagnosis of pulmonary mucormycosis.

**Table 1:** Clinical presentation of coronavirus disease 2019-associated mucormycosis.

Presentation	*n*=95
Headache	56
Nasal congestion and pain/blackening	18
Facial swelling and pain	15
Gum swelling and toothache	44
Eye swelling, red eye, visual complaints	18
Fever	4
Critical illness/diabetic ketoacidosis	0

Among the 96 cases, 53 had pre-existing diabetes, diabetes was diagnosed during COVID-19 in 37 cases, and the remaining six cases had no history of diabetes. Mean blood glucose level at admission was 236 (SD 82) mg/dL for patients with mucormycosis. Blood glucose was analysed with respect to extent of surgery, time to presentation, oxygen requirement and CT severity score. Mean glucose level at admission was statistically correlated with CT severity score (Spearman rho 0.248, *P*=0.014).

Steroid usage is shown in Table 2. The median duration of SARS-CoV-2 to mucormycosis diagnosis was 25 days [mean 25 (SD 14.1) days], and was shorter in patients without steroid exposure (median 31 days) compared with those who had received steroids. The median intervals were 18, 25 and 24 days for methylprednisolone, dexamethasone, and dexamethasone + methylprednisolone, respectively. This difference was significant (F=2.9, *P*=0.037). Post-hoc analysis showed that methylprednisolone intake during COVID-19 was significantly associated with a shorter time to the onset of mucormycosis compared with other groups [Tukey HSD mean 12.5 (SD 4.2) days; *P*=0.029] (see Supplementary Material 3).

**Table 2:** Use of steroids in coronavirus disease 2019-associated mucormycosis.

Type of steroid	Number of patients (*n*=95)	Median duration (days)
Methylprednisolone alone	20	14
Dexamethasone alone	8	7
Both methylprednisolone and dexamethasone	44	14
No glucocorticoid exposure	23	-

**Figure 4:**
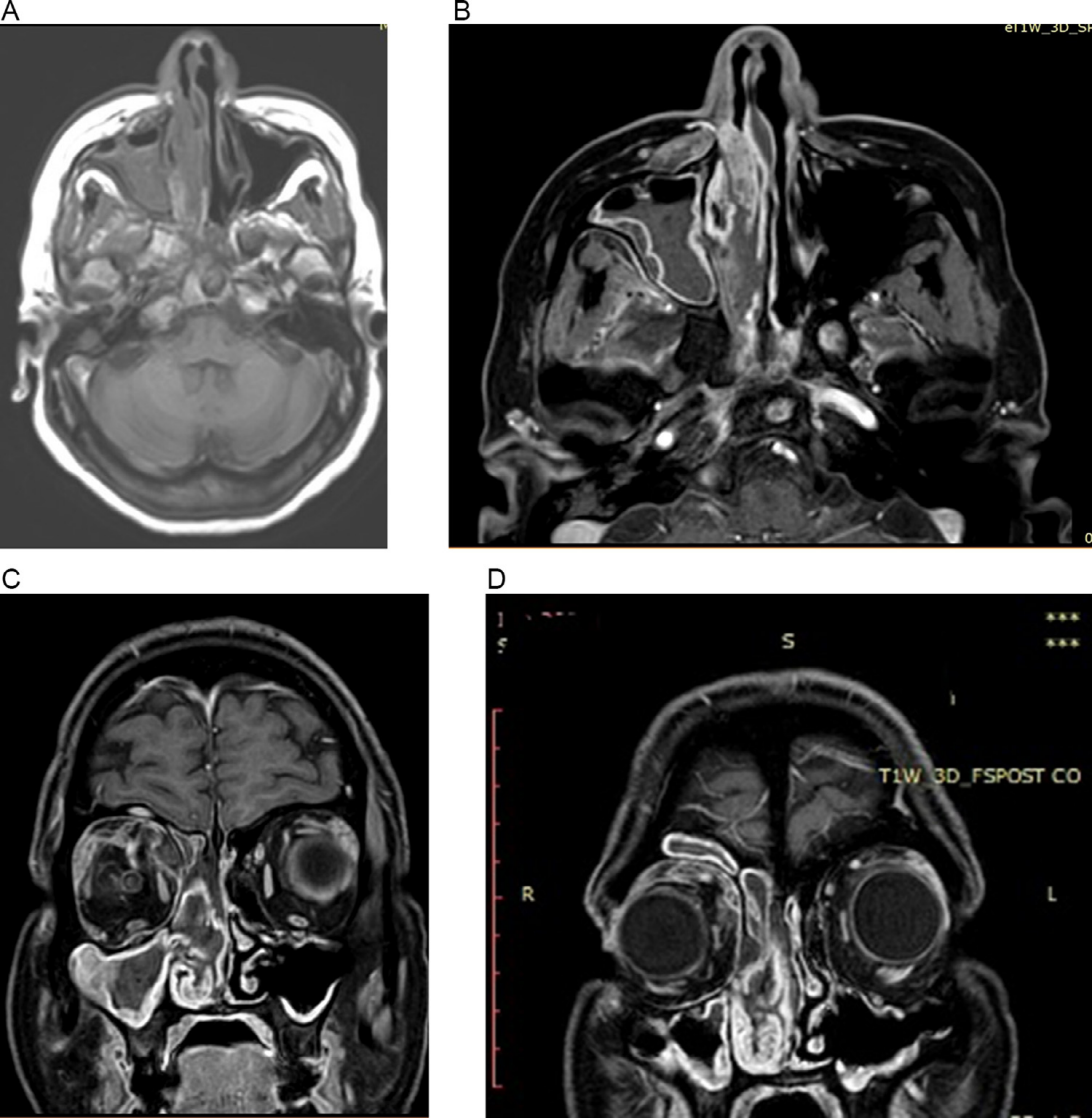
Magnetic resonance imaging (MRI) of coronavirus-associated mucormycosis. (A) T1WI axial section showing hypointense right maxillary sinus and right nasal cavity. (B) Post contrast MRI axial section showing enhanced inflamed mucosal lining of right maxillary sinus and nasal cavity. (C) Post contrast MRI coronal section showing retro-orbital involvement. (D) Post contrast MRI coronal section showing inflamed mucosal lining of right frontal sinus.

All patients had maxillary sinus involvement on CT/magnetic resonance imaging reports (Figure 4). Of these, 14 patients required simple surgery in the form of nasal debridement, and 81 patients required wider complete or partial maxillectomy, eyeball exenteration and repeated debridement. The median follow-up was 18 days. One patient could not be followed-up. As such, 95 patients were included in the statistical analysis of factors predicting extent of surgery. Two of these 95 patients died, and the rest were recovering and stable. Both deaths had diabetes, required ICU admission, and had received dexamethasone + methylprednisolone during COVID-19. With only two deaths, statistical analysis of factors predicting mortality was not possible. As such, binary logistic regression was used to identify factors predicting morbidity. Of the factors analysed, only ICU admission during COVID-19 (*P*=0.031) was negatively predictive of extended surgery. Hyperglycaemia, history of diabetes mellitus and steroid exposure were not associated with the need for extended surgery. No cases presented with diabetic ketoacidosis (DKA). The characteristics of clusters are presented in Tables 3 and 4.

**Table 3:** Cluster phenotype description.

	Cluster 1/COVID-19-associated mucormycosis and diabetes	Cluster 3/COVID-19-associated classical mucormycosis	Cluster 2/COVID-19-induced mucormycosis
Steroid exposure	+++	++	-
Pre-existing diabetes	+	+++	+
Blood glucose (mg/dL)	150–200	200–400	200–300
Severity of COVID-19	++	+++	++++
Atherosclerosis	++	+	++
Pathophysiology	Likely to have active COVID-19/steroid use/excessive dosage	Use of antibiotics/immunosuppression due to critical illness	COVID-19-associated small vessel thrombosis
Prognosis	Good	Bad, still better than non-COVID mucormycosis	Good

COVID-19, coronavirus disease 2019.

**Table 4:** Characteristics of different cluster groups.

	Total CAM (*n*=95)	CADM (*n*=44)	CACM (*n*=28)	CIM (*n*=23)
Mean age (years)	49.3	49.73	51.71	45.7
M/F	68/28	32/12	20/8	15/8
Clinical presentation				
Headache	56	20	17	19
Nasal congestion and pain/blackening	18	7	2	9
Facial swelling and pain	15	4	9	2
Gum swelling and toothache	44	23	14	7
Eye swelling, red eye, visual complaints	18	4	11	3
Fever	4	0	4	0
Critical illness/diabetic ketoacidosis	0	0	0	0
Site of involvement				
Maxillary sinus involvement	96	44	28	23
Ethmoid and/or sphenoid sinus involvement	68	34	23	11
Jaw and mandibular involvement	24	9	3	12
Orbital involvement	4	1	3	0
Hypertension present	10	3	5	2
Severity of COVID-19 (mild/moderate vs severe)	23/72	21/23	0/28	2/21
Mean CT severity score	5.05	5.5	6.07	4.1
Pre-existing diabetes/diabetes diagnosed after COVID-19/non-diabetic	40/50/5	7/32/5	28/0/0	5/18/0
Mean blood glucose on admission (mg/dL)	236	193	286	266
Use of steroids				
No steroids	23	0	0	23
Methylprednisolone	20	12	8	0
Dexamethasone	8	5	3	0
Both methylprednisolone and dexamethasone	44	27	17	0

COVID-19, coronavirus disease 2019; CAM, coronavirus disease 2019-associated mucormycosis; CADM, COVID-19-associated diabetes and mucormycosis; CACM, COVID-19-associated classical mucormycosis; CIM, COVID-19-induced mucormycosis; M, male; F, female; CT, computed tomography.

**Figure 5:**
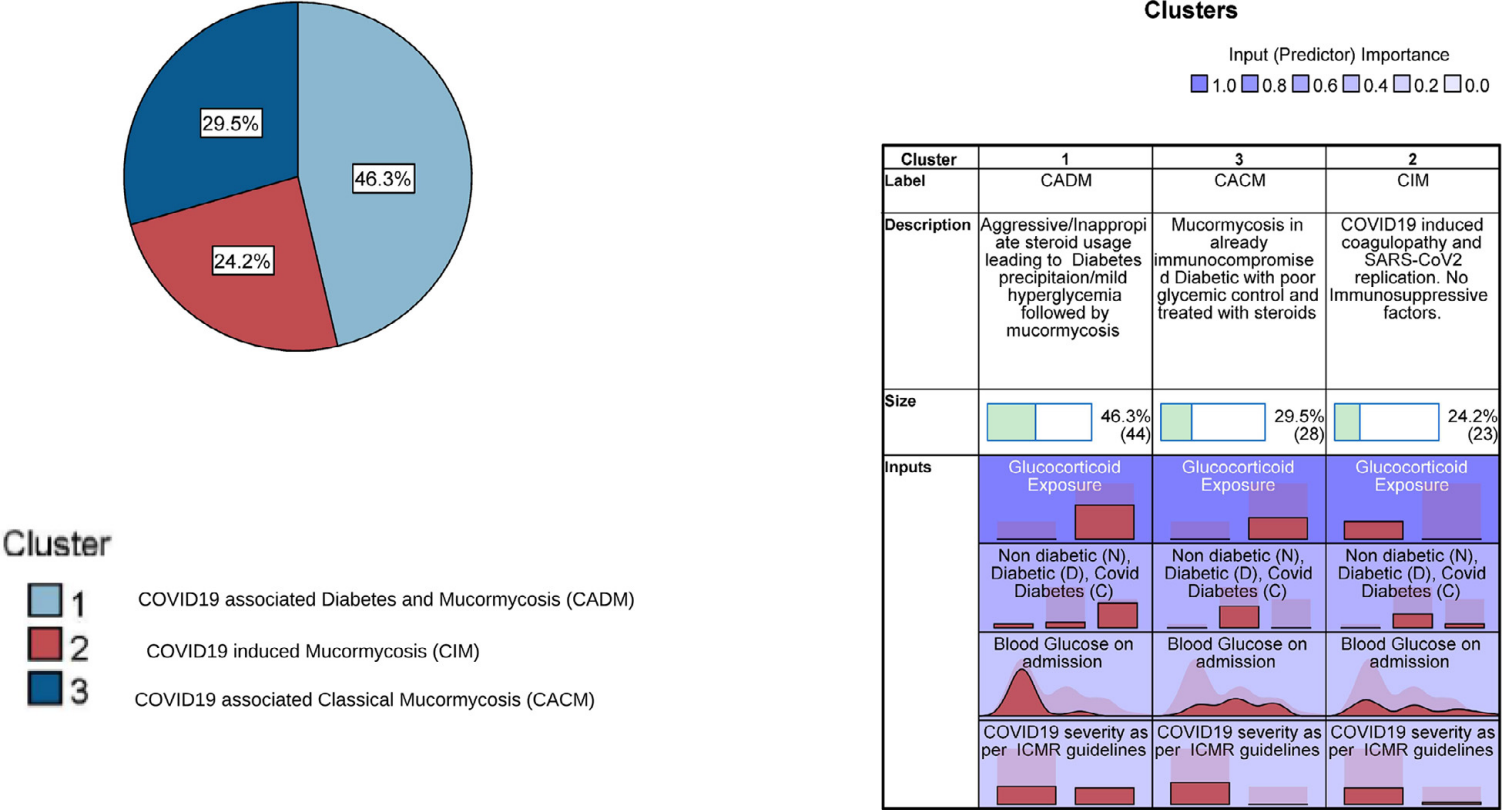
Cluster analysis. COVID-19, coronavirus disease 2019; SARS-CoV-2, severe acute respiratory syndrome coronavirus 2; ICMR, Indian Council for Medical Research.

**Figure 6:**
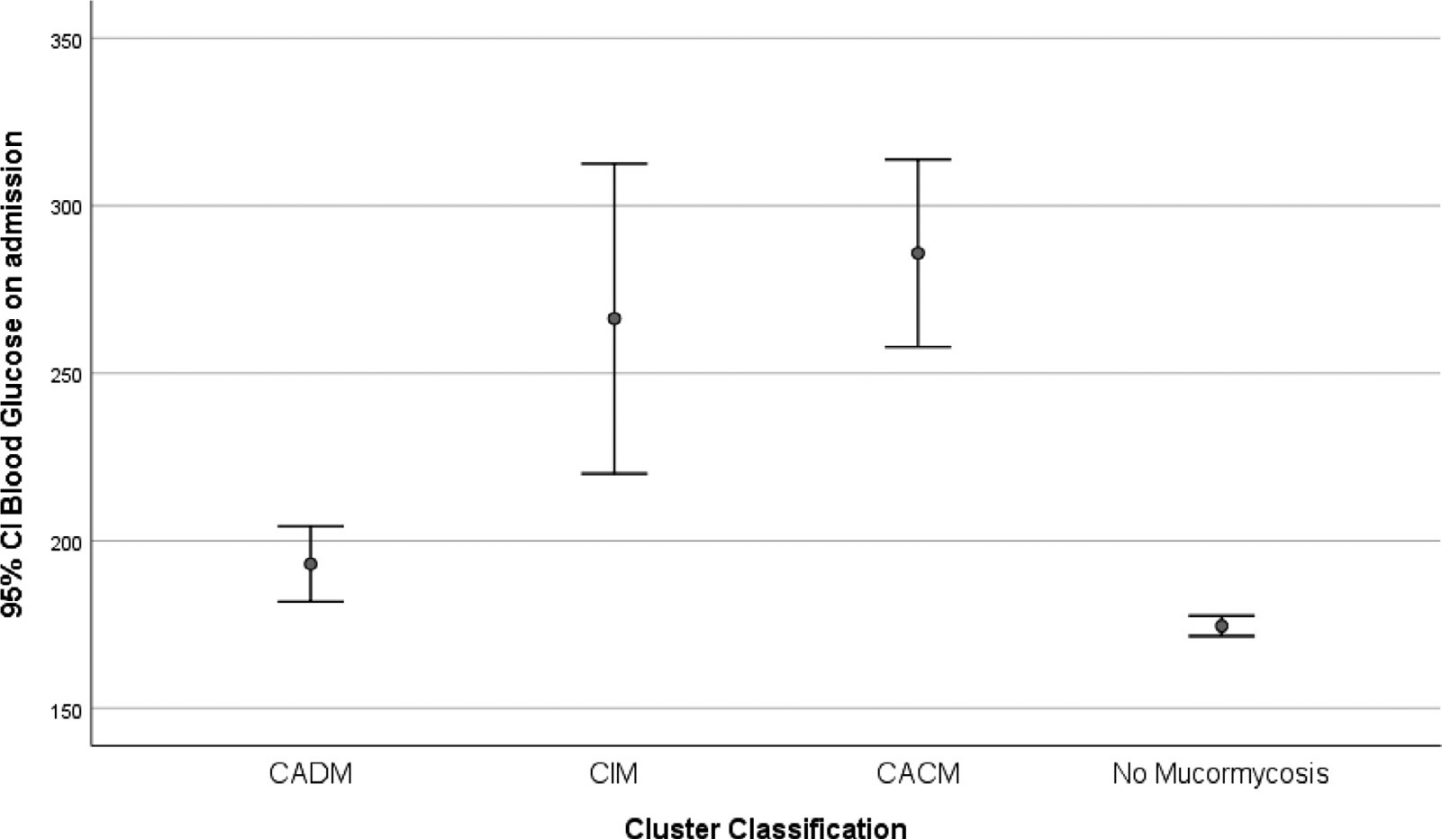
Comparison of blood glucose levels on admission of three clusters of patients with mucormycosis [coronavirus disease 2019 (COVID-19)-associated diabetes and mucormycosis (CADM), COVID-19-associated classical mucormycosis (CACM), and COVID-19-induced mucormycosis (CIM)] with those of 884 patients with COVID-19 admitted to COVID Care Hospital Chandrapur during the same period. CI, confidence interval.

Of the three clusters defined (Figure 5), Cluster 1 (46.3 %) and Cluster 3 (29.5%) had steroid exposure (100% in each), while Cluster 2 (24%) did not have steroid exposure (0%). Mild cases of COVID-19 were placed in Cluster 1. The mean blood glucose level was 193, 286 and 266 mg/dL in Clusters 1, 3 and 2, respectively. All cases placed in Cluster 3 had pre-existing diabetes (100%), while the majority of cases in Cluster 1 (71%) were diagnosed with diabetes mellitus during COVID-19. The mean blood glucose level at admission was 173.2 (SD 12) mg/dL for patients without mucormycosis. Tukey HSD post-hoc analysis showed that this was lower compared with Cluster 1 (193 mg/dL), but this difference was not significant (*P*=0.058). However, it was significantly lower than Cluster 3 (173 vs 286 mg/dL; *P*<0.001) and Cluster 2 (173 vs 266 mg/dL; *P*<0.001) (Figure 6).

## Discussion

### Epidemiology of CAM

The population incidence of mucormycosis in the pre-COVID era showed an increase over decades. It has been reported to be 1.2–1.7 per million in Western countries (Prakash and Chakrabarti, 2019). In Chandrapur and adjoining regions, there have been few cases of mucormycosis, most of which have occurred in patients with severe immunosuppression (Quraishi et al., 2020; Sagar et al., 2021). Frequency of mucormycosis at autopsy has been reported to be 6.3–33 per million hospitalized patients in tertiary hospitals (Prakash and Chakrabarti, 2019; Guinea et al., 2017). In comparison, the present study found that the prevalence of mucormycosis was 710 per million patients hospitalized with COVID-19. This points to factors related to the management of COVID-19, and the disease itself being causative. The prevalence of CAM in this study was 2300/million COVID-19 infections, which is at least 10-fold higher compared with the prevalence of mucormycosis in transplant recipients. The incidence of CAM in patients with COVID-19 is higher than that of mucormycosis in the general population. It is also several fold higher than that of mucormycosis in organ transplant recipients and patients with diabetes mellitus. This study found much lower prevalence (0.07% vs 0.27%) and mortality (2% vs 45%) rates compared with rates reported by Patel et al. (2021) from India. The differences can be attributed to referral bias, as the study by Patel et al. (2021) was retrospective and was carried out in tertiary hospitals. The present finding of higher male preponderance has been described previously for non-COVID mucormycosis and CAM (Prakash and Chakrabarti, 2019; Patel et al., 2021; Singh et al., 2021), and is likely due to the fact that testosterone increases expression of glucose regulated protein (GRP78), a stress protein crucial for hyphal invasion by *Mucorales* spp. (Ibrahim et al., 2020).

### Clinical presentation

Headache and orbital complaints were common, while fever was rare in this study. Previous case series on mucormycosis have described fever as a common presentation (Hingnikar et al., 2019; Quraishi et al., 2020; Sagar et al., 2021). The difference may be due to a debilitated general state in the former studies, and a peculiar interaction between COVID-19, steroids and hyperglycaemia inducing fungal invasion in the present study. SARS-CoV-2-induced transient T-cell suppression in the presence of broad-spectrum antibiotics, and the use of steroids may also explain the lack of fever. All of the patients in the present study had maxillary involvement. This is in contrast to previous CAM series (Patel et al., 2021; Sharma et al., 2021; Singh et al., 2021), which included substantial numbers of ethmoid, orbital and pulmonary mucor cases; this may suggest referral bias. Maxillary sinus is the most common site of non-invasive fungal infection, and is a likely conduit of fungal spores in otherwise healthy states.

### Role of steroids

While inappropriate use of glucocorticoids has been implicated previously in CAM, this study found that a sizable percentage (29.5%) of cases of CAM had not received steroids. Cases without steroid exposure were likely to present late. Glucocorticoids predispose to fungal angioinvasion by impaired neutrophil margination and repression of adhesion factors (Reusch et., 2021). In particular, methylprednisolone was found to be associated with earlier CAM presentation. Methylprednisolone impairs conidial phagocytosis by neutrophils, and predisposes to invasive fungal infection (Simitsopoulou et al., 2015). Due to perceived higher clinical efficacy against COVID-19, methylprednisolone has been widely used, in comparison with dexamethasone in India (Ranjbar et al., 2021). Whether the use of methylprednisolone per se could have contributed to increased prevalence of CAM in India remains speculative.

### Role of hyperglycaemia

In agreement with previous studies (Lu et al., 2021), the present study found strong correlation between CT severity score and blood glucose level at presentation. Mean blood glucose level at presentation (236 mg/dL) was higher than that described after severe COVID-19 (170 mg/dL) (Bode et al., 2020). However, it was much lower than levels at which mucormycosis occurred in pre-COVID times (Hingnikar et al., 2019; Quraishi et al., 2020; Sagar et al., 2021). In fact, the blood glucose levels of patients hospitalized with COVID-19 who did not develop mucormycosis did not differ from those in the COVID-19-associated diabetes and mucormycosis (CADM) cluster (see below). In the other CAM clusters [COVID-19-associated classical mucormycosis (CACM) and COVID-19-induced mucormycosis (CIM)], antecedent hyperglycaemia seemed to play a clear role in the cause of mucormycosis. Elevated blood glucose can increase viral replication exponentially by increasing glucose in the pulmonary airway surface liquid. The prevalence rates of undiagnosed diabetes and poor glycaemic control among patients with diagnosed diabetes are high in India. Even in patients with diagnosed diabetes, glycaemic control is poor. Non-CAM cases in this study had higher blood glucose levels that those described in other countries. However, the present findings for the CADM subtype call for the need to recognize factors above and beyond hyperglycaemia in the pathogenesis of CAM. None of the patients in this study had DKA, in contrast to the pre-COVID era.

### Role of critical illness

Patients admitted to the ICU were likely to undergo less extensive surgery, and this was independent of blood glucose level and steroid exposure. However, factor analysis did not find that ICU admission was a defining factor in cluste categorization. It is likely that patients admitted to the ICU received better care, mucormycosis was identified at an early stage, antifungal was administered early, and anticoagulant (low-molecular-weight heparin) was given. All of these factors may have reduced the severity of mucormycosis, and contributed to the need for less extensive surgery.

### Prognosis

Mucormycosis associated with diabetes, especially DKA, has a far better prognosis compared with mucormycosis associated with neutropenic states such as haematological malignancy. This is likely because underlying conditions such as DKA and hyperglycaemia can be optimized rapidly (Deutsch et al., 2019). The better-than-expected prognosios of patients with CAM in the present study can be explained similarly. The severity of COVID-19 is likely to decrease within 2 weeks in most survivors. This, along with cessation of steroids, may lead to the excellent overall prognosis in these patients.

### Classification and implication

Exploratory analysis for CAM classification was based on factors implicated as necessary for fungal mucosal invasion: diabetes, hyperglycaemia at mucormycosis presentation, use of glucocorticoid receptor agonists, and COVID-19. Type of diabetes (pre-existing, occurred during COVID-10/no diabetes) and hyperglycaemia at presentation were considered separately. A history of diabetes is suggestive of the presence of long-standing atherosclerosis, while hyperglycaemia at presentation is suggestive of metabolic milieu necessary for fungal proliferation. The use of glucocorticoids is representative of a qualitative defect in neutrophils (Table 3).

Cluster 1 (CADM) included the majority of cases. These subjects received steroids during COVID-19, followed by hyperglycaemia and diabetes precipitation, and had uncontrolled hyperglycaemia for some days. There was no predilection for orbit or lower jaw involvement. These patients had mild-to-moderate COVID-19, and aggressive glucocorticoid therapy precipitated diabetes and invasive fungal sinusitis. Most cases of active COVID-19 with mucormycosis were seen in this subgroup. These cases require single debridement and have an excellent prognosis. Control of hyperglycemia in this group – even for a few hours – can yield favourable results. Notably, this is the only subgroup in which mild cases were seen. This may point to inappropriate use of steroids.

Cluster 3 (CACM) consisted of the majority of cases with pre-existing diabetes. These subjects had longstanding uncontrolled diabetes, and severe COVID-19 followed by mucormycosis which required extensive surgery. These subjects were likely to have systemic involvement, orbital involvement and had a higher mortality rate. Blood glucose during mucormycosis admission for this cluster was the highest of the three subgroups (approximately 300 mg/dL), which is suggestive of uncontrolled diabetes. Clinically, this resembles mucormycosis described previously in patients with and without COVID-19. CACM is likely to be over-represented in studies from tertiary care hospitals. The only deaths that occurred in this study were patients in this subgroup. Aggressive surgery and antifungal therapy should mainly be directed at this group.

Cluster 2 (CIM) consisted of patients in whom a high SARS-CoV-2 load appeared to precipitate mucormycosis. These patients had not received steroids, did not have a history of diabetes, and had no risk factors for mucormycosis. Blood glucose levels were not significantly different from patients without mucormycosis. However, all subjects had severe COVID-19 and there was a predilection for jaw involvement (Table 4), including osteonecrosis of the jaw. The thrombotic response to SARS-CoV-2 may play a central role in causing fungal invasion in this subgroup.

This classification has practical implications for the management of patients with CAM. Current guidelines on CAM management bundle it into one entity. Appropriate triaging is needed given the highly heterogeneous nature of CAM. CADM and CIM may not require aggressive management, apart from hyperglycaemic control and simple debridement, whereas CACM should be managed aggressively. Antiplatelets/anticoagulants are likely to be the optimal preventative strategies for CIM.

Postulated CAM hypothesis

Identification of a phenotype without steroid exposure and associated with increased severity of COVID-19 points to a role played by the disease itself in the causation of mucormycosis. Expression of GRP78 is increased exponentially in patients with COVID-19 (Sabirli et al., 2021). Also, GRP78 itself plays a role in SARS-CoV2 internalization by interaction with the spike protein (Ibrahim et al., 2020). Thus, the GRP78–SARS-CoV-2 interaction occurs in a feed forward manner. **78-kDa glucose-regulated protein** GRP78 expression is increased four-fold in hypoxia. (Ibrahim et al., 2019) Thombosis of branches of the maxillary artery may induce intense mucosal hypoxia and acidosis. The Delta variant of SARS-CoV-2 has a propensity for arterial thrombosis (Vaidyanathan, 2021). Combined with steroid-induced neutrophil dysfunction, this may lead to fungal angioinvasion. This hypothesis also explains the excellent prognosis of CAM. Once COVID-19 resolves, GRP78 levels come down, blood supply and immunosuppression recover fast, and there is rapid clinical improvement. A certain level of hyperglycaemia seems to be essential for mucormycosis, but this plays a minimal role in recovery and final outcome. Steroid exposure is facilitatory in earlier precipitation and causation in susceptible patients, but it is not essential in the pathogenesis of CAM. The use of methylprednisolone may accelerate and possibly precipitate CAM. It is tempting to speculate that methylprednisolone may lead to increased GPR78 expression as glucocorticoid receptor agonists differ in transactivation profiles.

### Limitations

Data from registries have inherent disadvantages, such as lack of uniformity in clinical assessment and documentation. Information about antibiotic type, exact dose of steroids, and inflammatory markers, specifically ferritin, was not available and could have had a bearing on the classification of CAM. More importantly, data on environmental exposure were not available, and this information could have explained why many patients with diabetes do not develop mucormycosis (i.e. CIM).

## Conclusion

CAM is heterogeneous in terms of clinical presentation, phenotype and likely aetiology. Exposure to steroids is associated with early presentation. Use of methylprednisolone is a precipitating factor. There are three subtypes of CAM, of which only CACM resembles mucormycosis described in the literature. Future studies on CAM management should focus on conservative management and the role of antithrombotic therapies.

## Funding

This research did not receive any specific grant from funding agencies in the public, commercial or not-for-profit sectors.

## Ethical approval

This study was reviewed and approved by the ethics committee of Government Medical College, Chandrapur (No. GMCC/PSM/341/2021). This study was an observational study. Informed consent was obtained from all patients.

## Author contributions

AK, NR and RS designed the study. NR and JN recruited the patients and obtained clinical data. DM and AD analysed biological and radiological samples. RS and DM performed statistical analysis. RS, JN and AK wrote the article. All authors reviewed and approved the manuscript.

## Data availability

The raw data are available and deposited in OSF. They are accessible on the following link upon request: shukla, ravindra (2021, June 23). COVID 19 Associated Mucormycosis. Retrieved from osf.io/7fp2j.

## Conflict of interest statement

None declared.

## Appendix Supplementary materials

Image, application 1

Image, application 2

Image, application 3
