# Supplementary material for: Insights from Surgically treated Post Covid Acute Invasive Fungal Rhino-Orbital sinusitis in Chandrapur Study (SPAROS): A Population Based study of Coronavirus Associated Mucormycosis (CAM) characteristics in India
Source: IJID Reg. 2022 Aug 24;5:21–9. doi: 10.1016/j.ijregi.2022.08.005 (PMC9398937; doi:10.1016/j.ijregi.2022.08.005)
Supplement: Supplementary file 1 [file mmc1.docx]

**List of hospitals which contributed cases**

1. Gmc Chandrapur
2. Civil Hospital, Chandrapur
3. Wasade Hospital
4. Astha Nursing Home
5. Mehra Referral Hospital
6. Civil Hospital, Warora
7. Civil Hospital , Brahmapuri
8. Christ Missionary Hospital
9. Community Health Centre Mul
10. Tonge Hospital
11. Gulwade Hospital
12. Rural Hospital, Ballarpur

**Inclusion criteria**

- SARS-CoV-2 rRT-PCR positive after 1 March 2021
- Fungal infection involving head/neck/face/orbit on basis of
  - fungal hyphae on KOH mount
  - histopathological diagnosis of mucosa from the region involved
  - supporting radiological CT/MRI

**Exclusion criteria:**

- Active malignancy
- Organ transplant
- Those on iatrognic glucocorticoids for reason other than COVID-19
- Those refusing consent
- Lost to follow-up (telemedicine/physical) for >1 month
