# Supplementary material for: Insights from Surgically treated Post Covid Acute Invasive Fungal Rhino-Orbital sinusitis in Chandrapur Study (SPAROS): A Population Based study of Coronavirus Associated Mucormycosis (CAM) characteristics in India
Source: IJID Reg. 2022 Aug 24;5:21–9. doi: 10.1016/j.ijregi.2022.08.005 (PMC9398937; doi:10.1016/j.ijregi.2022.08.005)
Supplement: Supplementary file 2 [file mmc2.pdf]

Case Record Form

Name of Hospital :

Name of Treating Clinician:

Name :

Age:

Sex:

Address :

Date of Admission in index hospital

Date of Surgery   (if more than 24 hours after admission)

Presenting complaint: (Multiple options can be selected)

Headache
Nasal congestion and pain/ blackening
Facial swelling and pain
Gum swelling and toothache
Eye swelling, red eye , visual complaints
Fever
Critical Illness/Diabetic Ketoacidosis (DKA)
Any Other

Date of rtPCR positivity :

Blood Glucose on admission:

History of Hypertension: a. Yes

b. No

If yes, list antihypertensive medication

History of Diabetes :   a. Previous Diabetes Mellitus

b. Diabetes Mellitus diagnosed during COVID19

c. No history of diabetes

List of Anti-Diabetic Medicines : (Multiple options can be selected)

a. Insulin

b. Sulphonureas

c. SGLT-2 inhibitors

d. DPP4 inhibitors

Surgery Performed: (in 200 words write in brief the procedure , how biopsy sample was obtained and complications (if any), any unsual observation)

Post Surgery Medication

Anti-biotics

Antu-fungal (Multiple options can be selected)

a.Amphotericin B dose and duration

b. Posaconazole dose and duration

COVID19 related information: (To be obtained from previous records, If records not available pharmacy receipts can be used to substantiate)

COVID Severity as per ICMR :

a. Mild COVID19

b. Moderate COVID19

c Severe COVID19

Oxygen Requirement:

Hospitalization Requirement:

ICU Requirement:

Use of Remdesivir:

Glucocorticoids Exposure a.Yes

b.No

Glucocorticoid used: (Multiple options can be selected)

Dexamethasone	Duration:	Dose
Methylprednisolone	Duration:	Dose
Hisone	Duration:	Dose

Follow UP information (Select any one)

Status:

In Hospital ,stable

In Hospital, Unstable

In Home, recovering

Dead

Glycemic Control:

Medications:

Any Complaint:
