# Supplementary figures and images for: Insights from Surgically treated Post Covid Acute Invasive Fungal Rhino-Orbital sinusitis in Chandrapur Study (SPAROS): A Population Based study of Coronavirus Associated Mucormycosis (CAM) characteristics in India
Source: IJID Reg. 2022 Aug 24;5:21–9. doi: 10.1016/j.ijregi.2022.08.005 (PMC9398937; doi:10.1016/j.ijregi.2022.08.005)

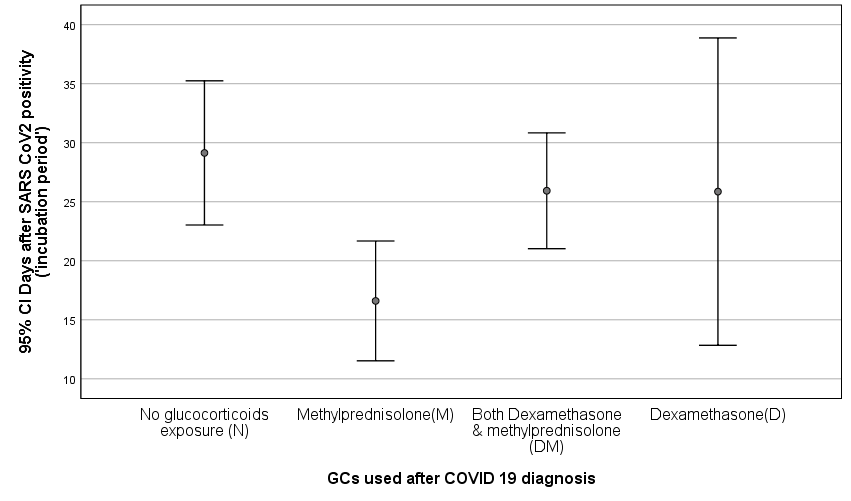

Supplement: Supplementary file 3 [file mmc3.docx]
